# *In vivo* CAR T cells and targeted gene delivery: A theme for the Pharmaceuticals and Medical Devices Agency Science Board to address

**DOI:** 10.3389/fmed.2023.1141880

**Published:** 2023-04-17

**Authors:** Rika Wakao, Ai Fukaya-Shiba

**Affiliations:** Center for Regulatory Science, Pharmaceuticals and Medical Devices Agency, Tokyo, Japan

**Keywords:** regulatory science, horizon scanning, drug development, CAR T cell, gene therapy, targeted gene delivery

## 1. Introduction

To ensure patients have timely access to innovative products, regulators must respond to evolving technologies by building up their expertise before reviewing the products with cutting-edge technology. Notably, new classes of therapeutics of unprecedented modality represent a regulatory challenge owing to the gaps in our knowledge. To accommodate emerging technologies, the Pharmaceuticals and Medical Devices Agency (PMDA) introduced “horizon scanning,” which involves systematic information gathering followed by activity prioritization (1). For the high-impact technologies that are difficult to assess under the existing regulatory standards, the PMDA Science Board, a committee of external experts, sheds light on significant points for the relevant area to ensure an optimal review and appropriate regulation (2). Herein, we would like to discuss the latest issue of the PMDA Science Board, “*In vivo* chimeric antigen receptor (CAR) T cells generation: An assessment of targeted gene delivery” (3). This is a regulatory effort not only to catch up with technological advancements but also to facilitate rational drug development. The purpose of this report is to inform the general readers about the regulatory challenges in the development of *in vivo* generated CAR T products and to assist foreign regulators and developers in following up discussions by the PMDA Science Board to support efficient development.

CAR T cell therapy is a cancer immunotherapy that has brought substantial benefits to patients with B cell malignancies (4). In addition to hematological malignancies, many efforts are ongoing to extend the CAR T cell approach to solid tumors and viral infections (5). However, personalized CAR T cell production is a bottleneck due to its complexity and resource requirements: T cells are extracted from patients *via* leukapheresis, and then these cells are genetically modified and expanded *ex vivo* at any eligible facility under current good manufacturing practices (6). Patient-derived starting materials may have intrinsic properties that affect CAR T cell manufacturing because of the disease state and prior treatment, leading to uncertainty in product completion. In addition, the production process normally takes several weeks, which is not feasible for rapidly progressing cancer patients who need immediate treatment. Another challenge for patients is to receive the standard preconditioning chemotherapy, which depletes endogenous lymphocytes before the infusion of the *ex vivo*-expanded CAR T cells. To overcome the barriers of the current *ex vivo* CAR T cell therapy, intensive research efforts are in place to generate CAR T cells *in vivo* through direct injection of the gene vector (7–9). *In vivo* CAR T cell generation could make this treatment immediately accessible to patients. However, efficient and highly selective gene delivery to target cells *in vivo* is challenging. Given that this issue is suitable for drug review and regulation, the PMDA Science Board selected this issue for the next technical subcommittee.

## 2. Current progress in CAR T cell generation *in vivo*

### 2.1. Lentiviral vectors (LV)

To confine the tropism of CAR-encoding LV to T cells, it is necessary to destroy natural receptor usage and fuse the binders which recognize T lymphocyte marker, such as CD8 or CD4, or use a non-covalent adapter (see below) (10–13). Human CD19-CAR T cells can be directly generated *In vivo* with LV targeting human CD8+ cells in mice (14). CD8-targeting LVs encoding a CD19-specific CAR exerted antitumor activity in immunocompromised mice with B cell lymphoma (15). The same group found that CD4-LV exerted much faster and more efficient tumor cell killing activity than that of CD8-LV alone or in combination (16). LV vectors targeting human CD3 have been described previously. It has been reported that CD3-LV induced T cell activation and proliferation, allowing for the *in vivo* generation of functional CAR T cells (17). Another study reported that a bispecific antibody recognizing both CD3 and the mutated envelope protein E2 mixed with LV conferred selectivity for transducing CD3 T cells, resulting in CAR T cells. These CAR T cells exerted antitumor activity *in vivo* (18).

### 2.2. Adeno-associated virus (AAV) vector

One report showed that human CAR T cells can be generated *in vivo* by injecting an AAV vector carrying the CD4 targeting CAR gene. In a humanized mouse model of T-cell leukemia, AAV generated a sufficient number of potent CAR cells *in vivo* to promote tumor regression (19).

### 2.3. Non-viral platforms

Given the international rollout of messenger RNA (mRNA)-based SARS-CoV-2 vaccines, non-viral vectors, including lipid nanoparticles (LNPs) and nanocarriers (NCs), have recently gained worldwide attention, stimulating broad efforts to extend this therapeutic platform to address numerous pathologies (20).

#### 2.3.1. T cell–targeted nanoparticles

A previous study reported that CD3-targeted NCs with plasmid DNA introduced leukemia-targeting CAR genes into T cells, thereby resulting in disease remission (21). However, there are some disadvantages to DNA transfection, such as genomic insertion and promoter dependency. Accordingly, in the follow-up study, the same group used *in vitro*–transcribed (IVT) mRNA. Repeated infusions of CD3- or CD8-targeted NCs with CAR or T cell receptor (TCR) mRNA induced a sufficient number of T cells to express tumor-specific CARs or virus-specific TCR (22). This resulted in disease regression in mouse models of human leukemia, prostate cancer, and hepatitis B-induced hepatocellular carcinoma.

Another study reported on the antifibrotic CAR T cells designed against fibroblast activation protein (FAP, a marker of activated fibroblasts): Injections of FAP-CAR-mRNA in CD5-targeted LNPs decreased the burden of fibrotic tissue and improved cardiac function in a mouse model of fibrosis (23).

With easier handling and well-timed administration at medical facilities, these NC platforms could potentially be therapeutic alternatives as the off-the-shelf reagents for a wide range of diseases.

It remains to be addressed whether nanoparticles (without T cell–targeting ligands) using modified lipid compositions can generate CAR T cells *in vivo* (24).

### 2.4. Others

In addition to infusion, implants have been reported to reduce the time for manufacturing CAR T cells: CAR-encoding retroviral particles have been incorporated into implantable scaffolds that were used to transfer human immune cells, leading to CAR T cell generation *in vivo* (25).

## 3. Discussion

Intensive efforts have been focused on engineering CAR T cells directly *in vivo* to bypass *ex vivo* manipulation. While these innovative products offer clinical opportunities, they also present regulatory challenges that the Science Board is going to address.

(1) Mitigating the risk of off-target transduction
A study reported that the unintentional transduction of a single leukemic B cell during anti-CD19 CAR T cell manufacture led to resistance against the CAR T cell therapy by masking the CD19 epitope, resulting in cancer relapse and the death of the patient enrolled in a phase 1 trial (26). To mitigate the risk of *ex vivo* manufacturing, in the approved procedure, the purity of T cells can be monitored by using in-process and quality control tests. In the absence of process control for regenerative cell products, the accuracy of targeted gene delivery is of prime importance for *in vivo* CAR T cell generation. Alternatively, there may be ways to use transient expression systems for indications other than cancer. The Science Board will discuss strategies to identify and mitigate risks for first-in-human clinical studies based on state-of-the-art technology.
(2) Rational vector design and related guidance
Vectors that selectively transduce or transfect therapeutically relevant cell types upon systemic administration are expected to reduce the required dose and toxicity. Receptor targeting is an approach to rational vector design. A defined cell surface receptor, such as CD3, CD4, CD5, or CD8, is targeted by both viral and non-viral vectors to achieve selective delivery to the intended cells. Given that synthetic nanoparticles with cell-surface receptor binders should harbor correctly oriented binders and proper three-dimensional structures to ensure the intended pharmacological activity with safety and efficacy, it is of prime importance to rigorously control the manufacturing process. In this regard, the FDA guidance on nanomaterials can be referenced (27). For viral vectors, Guidelines for Ensuring the Quality and Safety of Gene Therapy Products provide principles for quality control, non-clinical studies, and clinical development (28). The Science Board will discuss the rational design of targeted gene delivery that is not covered by the existing guidelines, including quality, targeting, biodistribution, dosing, immunogenicity, and expression kinetics.
(3) Class effects on safety that are observed in relevant vector platforms
Cytokine release syndrome and immune effector cell-associated neurotoxicity are major toxicities associated with CAR T cells. In a preclinical model, the *in vivo* generation of CD19-CAR T cells resulted in B cell depletion and signs of cytokine release syndrome (14). Serious adverse events have been reported in AAV clinical trials, including hepatotoxicity, neurotoxicity, and thrombotic microangiopathy (29). Can non-viral vectors reduce hepatotoxicity and other adverse events associated with AAV vectors? For each vector platform, the Science Board will consider the class effects on safety.

**Figure 1:**
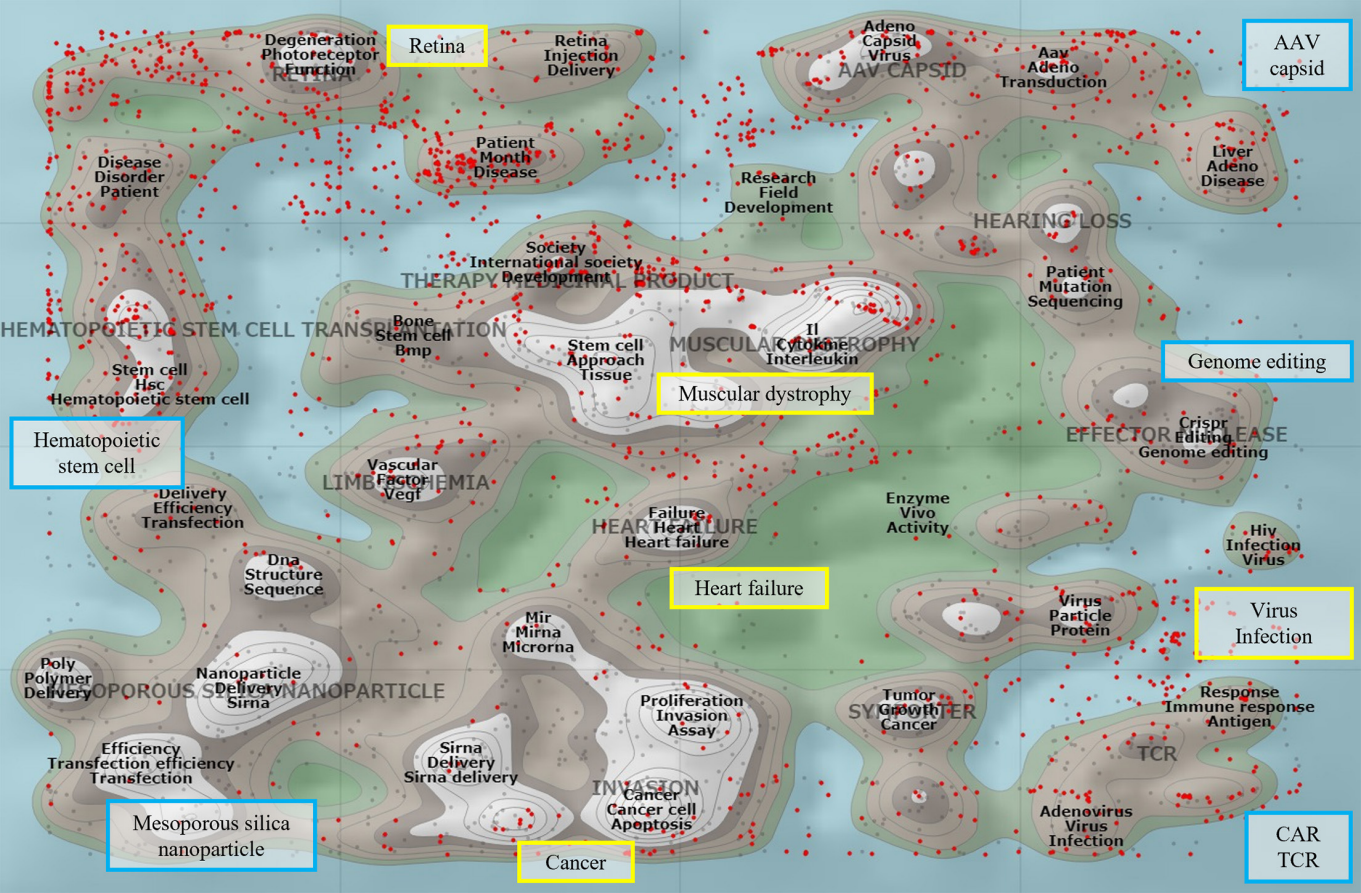
The literature landscape of gene therapy. The ThemeScape map, an analytical tool in Derwent Innovation (Clarivate Analytics), conducts text-mining-based literature clustering. Common conceptual terms are displayed on a two-dimensional map which shows the relative relationship of one article to another, with peaks representing a concentration of documents. The map comprising 14,770 publications was visualized by the extracted terms from the titles and/or abstracts relevant to the gene therapy articles in Web of Science between 2015 and 2022. It depicts high-density clusters for the disease areas (rectangle bordered in yellow) and technological domains (rectangle bordered in blue), such as chimeric antigen receptor (CAR), T cell receptor (TCR), genome editing, AAV capsid, and mesoporous silica nanoparticles. The red dots represent the articles identified with the terms “clinical or trial.”

The elaborate assessment of targeted gene delivery by the Science Board would apply not only to CAR T cell generation but also to genome editing, which is conventionally manipulated *ex vivo*. Figure 1 shows the literature landscape of gene therapy pertinent to the field. TCR T and CAR T articles form peaks, and the peak of genome editing literature is found in the vicinity. These fields are within the scope of the discussion by the Science Board, which will present points to consider for entering the first-in human clinical study. The outcome report will streamline the review process and the overall development of these fields.

From an international point of view, global regulators cooperate in the International Coalition of Medicines Regulatory Authorities (ICMRA), where horizon scanning is one of the focuses in which the PMDA is participating (30). In a recent publication on horizon scanning by the European Medicines Agency, a subset of 25 technological trends has been identified, many of which overlap with what the PMDA is also paying attention to (31). Using the ICMRA network, reports from the PMDA Science Board will help facilitate safe and timely access to innovative medicines globally.

## Author contributions

RW conceived the idea and wrote the manuscript. AF-S performed the analysis of the available evidence. All authors contributed to manuscript revision, read, and approved the submitted version.
